# Sticky Floor, Broken Ladder, and Glass Ceiling: Gender and Racial Trends Among Neurosurgery Residents

**DOI:** 10.7759/cureus.18229

**Published:** 2021-09-23

**Authors:** Hamza Maqsood, Shifa Younus, Sadiq Naveed, Amna Mohyud Din Chaudhary, Muhammad T Khan, Faisal Khosa

**Affiliations:** 1 Internal Medicine, Nishtar Medical University, Multan, PAK; 2 Psychiatry, Hartford Hospital - Institute of Living, CT, USA; 3 Psychiatry, Nishtar Medical College and Hospital, Multan, PAK; 4 Neurology, Charleston Area Medical Center, Charleston, USA; 5 Radiology, Vancouver General Hospital, Vancouver, CAN

**Keywords:** neurosurgery, residents, gender disparity, racial disparity, under-represented minorities, equity, diversity

## Abstract

Introduction

Diversity and equity in academic medicine are critically important in improving healthcare standards and patient-related outcomes. Gender and racial disparities are some major challenges faced by the health system. This article reviews the gender and racial trends among residents of neurosurgery in the United States (US).

Methods

We retrospectively analyzed the data extracted from the Accreditation Council for Graduate Medical Education (ACGME)’s annual Data Resource Books from 2007 to 2019. ACGME cataloged gender as men and women and race/ethnicity was categorized as White/non-Hispanic, Asian or Pacific Island, Hispanic, Black/non-Hispanic, Native American/Alaskan, others, and unknown. Counts, proportions, relative, and absolute percentage changes were calculated to highlight trends in resident appointments over time and across the specialty of neurosurgery.

Results

The number of female residents increased steadily from 10.6% in 2007 to 19.3% in 2019; with an absolute increase of 8.74%, a relative increase of 63.9%, and a simultaneous decrease in male residents. When averaged across the nine-year study period, 51% of the study sample was White (non-Hispanic), followed by Asian/Pacific Islanders at 15.2%. The representation of Hispanics was 4.3%, Black/African Americans were 4.5%, Native Americans/Alaskans were 0.2%, and others were 8% of the total study population.

Conclusion

Our study concludes that gender and racial disparity persist within the neurosurgery residency training programs in the US. Concrete efforts at all academic levels are needed to provide greater support for the females and for the careers of underrepresented minority (URM) trainees to ensure their increased representation in neurosurgery.

## Introduction

The United States (US) is the third-most populous country globally, with an estimated population of 331 million as of December 2020, with males constituting 49.4% while females comprise 50.6% of the total population [1]. According to the US Census Bureau's estimates, population growth is fastest among minorities, with 50% of US children under 18 belonging to ethnic minorities [2]. The foreign-born population doubled from almost 20 million in 1990 to over 45 million in 2015, thus representing one-third of the population increase [3]. On the other hand, White/Caucasian were 73%, Blacks/African Americans 12.7%, Asians 5%, American Indians/Native Alaskans 0.8%, Native Hawaiians/Pacific Islanders 0.2%, and other populations comprised 2% of the total US population [4].

With the increasing population of minorities in the US, the variegation of health faculty and professionals is crucial to improve health standards and patient outcomes [4]. A diverse healthcare workforce improves healthcare access for minorities, facilitates better communication with patients, and provides culturally sensitive quality care for underrepresented minorities (URMs) [5]. It also enhances innovation and productivity, leading to improved morale of the healthcare workers and a better working environment [6,7]. Racial disparity is a threat to every person's physical, emotional, and social well-being in a society that allocates privilege based on race [8]. Despite ongoing efforts for equity, diversity, and inclusion, disparities are prevalent in academic disciplines, professional societies, and editorial boards of medical journals [9-13].

Similarly, women are underrepresented across all professions, disciplines, and career paths. This inequality manifests as early as the first promotion and progressively becomes more apparent at subsequent levels of career advancement [9]. Women are far less represented at higher academic levels [10]. A study done by Saleem et al. on neurology residents showed that females have very little representation at higher academic ranks [10]. Although women now constitute >50% of current medical school graduates in the United States, surgical specialties have one of the largest gender gaps [14,15]. In the US, it was reported that 14% of women choose to go into a surgical subspecialty compared to 33% of men [13]. As such, women represent only 12% of neurosurgeons in the United States [16].

Several factors, including isolation, lack of camaraderie among current residents, discrimination, communication barriers, greater debt burden, work-life imbalance, lack of mentorship, and lack of females and minorities role models, are some of the factors contributing to gender and racial disparities in the healthcare system [16-18]. Medical organizations like the American Medical Association (AMA), the American Association of Medical Colleges (AAMC), and the National Medical Association (NMA) have made task forces to diversify representation resulting in a more creative, productive, innovative, and egalitarian environment. Despite all the efforts, there has been slow progress towards equity, diversity, and inclusion in the academic neurosurgery workforce over the past decade [19].

This study investigated the gender and racial disparity trends in neurosurgery residents across the US between 2007 and 2019.

## Materials and methods

Data extraction

Two team members (HM, SY) independently extracted the data from the Accreditation Council for Graduate Medical Education (ACGME)'s annual Data Resource Books. In this paper, we extracted the data for the residents in the discipline of neurosurgery. The data for gender distribution are available from 2007 to 2019, whereas the data for races/ethnicity are available from 2011 to 2019. The data were extracted into the Microsoft Excel sheets. The third team member (SN) reviewed the extracted data for accuracy and resolved any discrepancies by discussion. Race/ethnicity was categorized as White/non-Hispanic, Asian/Pacific Islander, Hispanic, Black (non-Hispanics), Native American/Alaskan, others, and unknown. Gender was categorized as male, female, and not reported.

Data analysis

We analyzed the data by gender and racial distributions and its temporal trends by year among neurosurgery residents using Statistical Package for the Social Sciences (SPSS), version 27 (IBM Corp., Armonk, NY). Counts, proportions, relative, and absolute percentage changes were calculated to highlight trends in fellows appointments over time and across the discipline of neurosurgery.

Ethical approval

Ethical approval was not needed since there was no involvement of human or animal subjects. Our team sought all the information from the publicly available data of the ACGME.

## Results

**Figure 1:**
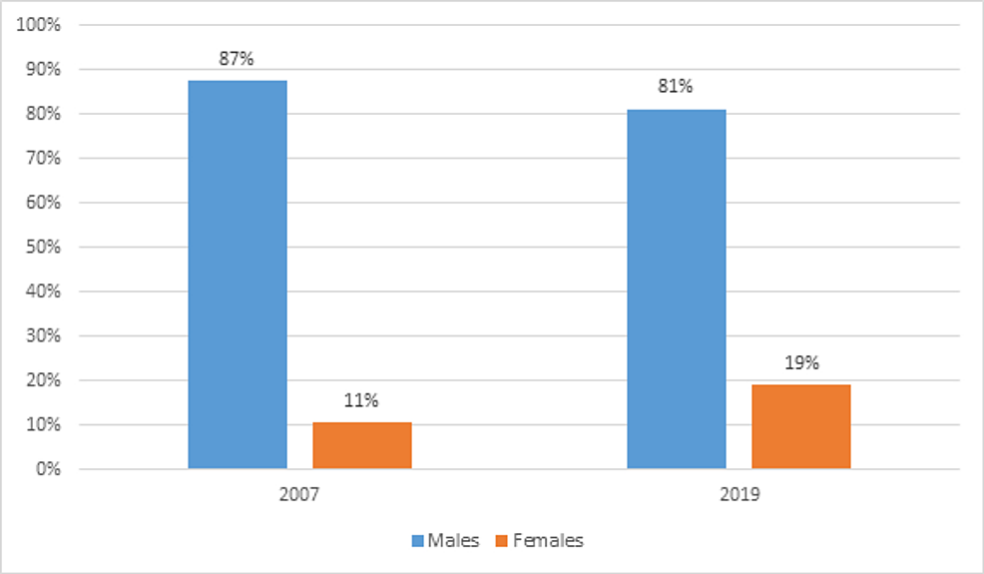
Gender differences at the beginning and end of our study period (i.e., 2007 to 2019).

The representation of females increased steadily with a relative increase of 8.74% from 2007 to 2019. However, males had a far greater representation as compared to females throughout the study period. In 2007, males accounted for 87.43% while females accounted for 10.56% of all residents in neurosurgery whereas, in 2019, males accounted for 81% while females accounted for 19.3% of all academic neurosurgeons (Figure 1).

As for the racial distribution, our study period ranged from 2011 to 2019. When averaged across the nine-year study period, 51% of the study sample was White (non-Hispanic), followed by Asian/Pacific Islanders at 15.2%. The representation of Hispanics was 4.3%, Black/African Americans were 4.5%, Native Americans/Alaskans were 0.2% and others were 8% of the total study population. For 17.2% of the fellows, the racial data were not known and was categorized as Unknown racial distribution. The absolute change in racial distribution was highest for Whites (+5.1) followed by Asian/Pacific Islander (+2.5), Hispanics (+1.5), Black/African Americans (+0.3), Native Americans/Alaskans (−0.12), and others (−1.6) (Table 1).

**Table 1:** Gender and racial differences as well as absolute changes at the start and end of our study period.

	2011 (%)	2019 (%)	Absolute change (%)
White	50.47	55.55	5.1
Asian/Pacific Islander	14.50	17	2.5
Hispanic	4.24	5.8	1.56
Black/African Americans	4.50	4.8	0.3
Native Americans/Alaskans	0.25	0.13	−0.12
Others	9.33	7.72	−1.61
Unknown	16.71	8.84	−7.87
	2007 (%)	2019 (%)	Absolute change (%)
Males	87.43	81	−6.43
Females	10.56	19.3	8.74

The yearly percentage of all neurosurgery residents by race and gender is shown in Table 2.

**Table 2:** Temporal trends for race and gender and absolute percentage change from the year 2007 to 2019.

	2007 (%)	2008 (%)	2009 (%)	2010 (%)	2011 (%)	2012 (%)	2013 (%)	2014 (%)	2015 (%)	2016 (%)	2017 (%)	2018 (%)	2019 (%)
White					50.4	49.4	50.3	49.8	49.8	49.9	50.7	49.8	55.5
Asian/Pacific Islander					14.5	15.4	15.1	15.2	15.4	15.2	14.1	14.3	17.1
Hispanic					4.2	4.4	4.2	4.3	3.8	3.7	3.6	4.2	5.8
Black					4.5	4.5	4.6	4.5	4.4	4.4	4.1	4.0	4.8
Native American/Alaskan					0.25	0.24	0.15	0.15	0.15	0.21	0.21	0.13	0.13
Others					9.3	8.1	8.6	8.2	7.8	7.1	7.0	7.1	7.7
Unknown					16.7	17.7	17.7	17.5	18.3	19.2	20	20.2	8.8
Male	87.4	86.2	86.0	84.1	80.3	82.1	83.0	81.5	82.0	81.2	81.3	82.0	81.0
Female	10.6	11.6	12.6	14.2	15.0	16.1	16.3	17.2	17.0	17.3	17.6	17.3	19.3
Not reported	2.0	2.1	1.3	1.6	2.0	1.7	1.4	1.2	1.2	1.1	0.9	0.6	0.5

## Discussion

Gender disparity

The number of female residents increased steadily from 10.6% in 2007 to 19.3% in 2019; with an absolute increase of 8.74%, a relative increase of 63.9%, and a simultaneous decrease in male residents. These findings are consistent with previous studies, both for neurosurgery and other surgical specialties [9,16,18]. Another study done by Woodrow et al. revealed that an estimated 13% of practicing surgeons and 6% of neurosurgeons in North America were women, while the number of female neurosurgical residents was approximately 12% [20]. In our study, despite a slower growth rate, the absolute number of female residents increased from 2007 to 2019. Our findings are also corroborated by existing literature that indicated a considerable growth for women's empowerment in the twentieth century. This remarkable growth began with the pioneer, Ruth Kerr Jakoby MD, who was the first woman to receive board certification from the American Board of Neurological Surgery (ABNS) in 1961. Since then, this number has grown significantly over the last 60 years, with over 200 ABNS-certified female neurosurgeons [21-23]. Despite this progress, female representation at the training level and academic ranks of neurosurgery are inadequate [9].

Several factors have been ascribed to the recruitment of residents in neurosurgery such as the United States Medical Licensing Examination (USMLE) scores. In a study, Durham et al. analyzed the results of 18 neurosurgery residency cycles and showed that USMLE Step 1 score and the applicant’s medical school ranking were the most important predictors of successfully matching in a neurosurgery residency program. Interestingly, despite being parallel in USMLE Step 1 scores and medical school ranking, female neurosurgery residency applicants are less likely to match into neurosurgery training than male applicants [24].

Deciding factors for choosing a residency have been examined throughout medical and surgical specialties. However, very few studies were focused only on female applicants. In 2007, Cole. et al identified influential factors including a friendly training environment, communication level among residents, variety, and the number of cases, and quality of relationships with attending physicians was reported. They concluded that women ranked programs with a greater percentage of women faculty higher [25].

Isolation, lack of camaraderie among current residents, poor quality of relationship with attending, decrease research opportunities, work-life imbalances, pregnancy, and parental leaves, unconscious bias and harassment, and lack of mentorship due to inadequate representation of females at higher academic tiers are some factors that can lead to racial and gender disparity [18,26]. Pregnancy and parental leave are among the most important issues to address. The obstacles to taking parental leave were a lack of universal policy, strain on the residency program, loss of training time, lack of flexibility, and lack of support from faculty or peers [27]. In a recent survey analysis of 347 female surgeons, 63% responded that an unmodified surgical residency program during pregnancy was perceived as unhealthy for either the female surgical trainee or the unborn child. Perhaps of most concern, approximately 40% of female surgeons considered leaving surgical residency, and 30% said that would discourage female medical students from opting for a surgical career [28]. Among neurosurgeons without children, long work hours were ranked as first by the majority of the respondents for not having children, followed by lack of partner support [29].

There is a need to develop a comprehensive plan to attract, retain and support the careers of women in neurosurgery. The representation of women at all levels of neurosurgery training, professional development, and organized neurosurgery leadership should be encouraged. The residency training programs should be encouraged to vigorously pursue diversity in their hiring practices. More female neurosurgeons should be encouraged to complete the nationwide leadership programs [9]. The Healthcare system should support paid parental leave with flexibility and adjustments in surgical training to protect the health of pregnant surgical trainees and their unborn children. There should be a zero-tolerance policy on bullying and harassment. Workshops could be offered to offer experiential learning on negotiating skills for academic or hospital contracts, leadership skills, research, and academic advancement. The medical student curriculum should be redesigned to attract prospective female neurosurgical trainees and to promulgate the importance of diversity at all levels of neurosurgery training, practice, and leadership [30]. Female medical students should be exposed to more neurosurgical elective experience. Acknowledging and publicizing the promotion of female neurosurgeons could motivate women in their early careers to aspire and prepare for leadership positions in the future [31].

Racial disparity

A similar trend was seen while comparing the representation of different races within the residency training programs of neurosurgery in the US. Throughout our study period, the White/Caucasian race was over-represented among all neurosurgery residents followed by Asian/Pacific Islanders. There can be several explanations for our findings. A previous study attributed the increasing Asian faculty representation to a parallel increase in the total population of Asians in the US [4]. Over the past two decades, the Asian population has been the fastest-growing racial or ethnic group in the US [32]. Several barriers are faced by URMs in getting a residency training spot and promotion in faculty, including a greater debt burden, limited communication skills, as well as racial prejudice and discrimination at the workplace [16,33]. The recruitment and retention of ethnic minorities are also affected by the lack of minority preceptors [34].

There are several factors that contribute to racial disparities beyond personal discrimination. Structural/institutional racism is as important as individual discrimination [35]. Structural racism especially towards the Blacks/African Americans reflects a system in which public policies, institutional practices, and other norms perpetuate racial group inequality. Most physicians are not explicitly racist and treat patients on an equal basis. However, the system in which they work is inherently racist [36]. Structural racism is insidious, and various studies document disparate outcomes for different races despite the best efforts of individual health care professionals [36].

The advantages of increasing the representation of URMs in neurosurgery are multi-faceted. First, it will lead to better communication with patients and would make health care more accessible and diverse [37]. Second, cultural variability will enable surgeons to diversify their health beliefs and improve compliance and prognosis [37,38]. Last, the promotion and retention of URM faculty would provide identifiable preceptors for minority students which will lead to increased recruitment of URMs medical graduates in neurosurgery [37].

Thus, to provide clinical care and conduct research that contributes to equity, we believe it is crucial to “center at the margins.” Centering at the margins signifies that we should re-anchor our academic and health care delivery systems, specifically, diversification of the workforce [8]. There is also a need for intensive and systematic educational campaigns to highlight the problem of disparities in the health care workforce. The awareness levels of the public and professional community, especially the medical community, must be raised [35]. A study reported that to reach racial and ethnic population parity, the US needs to double the number of Black/African Americans and Hispanic first-year residents and triple the number of Native American residents. White/Caucasian first-year residents would need to be reduced by two-fifths and Asians by two-thirds [38]. But current trends show that these goals will remain elusive for the foreseeable future.

The findings of this study indicate that further research is needed into the multifactorial reasons contributing to the decreased representation of women and URMs in residency training programs in Neurosurgery.

Limitations

Our study has its share of limitations. This study focused on data from neurosurgery residents within US residency programs and thus may have limited generalizability to other specialties and other parts of the world. Our study utilized a dataset that describes gender in a binary fashion. Finally, our study did not explore the effects of intersectional identity or being both a gender and a racial minority, such as female Hispanic or female Black/African American residents.

## Conclusions

Our study concludes that equity, diversity, and inclusion remain elusive in neurosurgery residency training programs. Efforts at all levels are needed to provide greater support for the careers of females and URM trainees, as well as faculty to ensure their representation at all levels of academic neurosurgery. We hope that the current policies at the level of medical institutions and associations will promote and encourage more women and URMs in choosing neurosurgery as their career choice. Further longitudinal studies are needed to evaluate the long-term definitive impact of all the implemented policies and initiatives in increasing the representation of gender and racial minorities.
